# Elevated serum glucosylsphingosine level in children with obesity: relation to plasma atherogenesis

**DOI:** 10.1038/s41366-025-02016-9

**Published:** 2026-01-22

**Authors:** Nouran Y. Salah, Dina Abdel Hakam, Fatma AbdEllah Abdullah, Marwa Samir Hamza, Eman Aly Ramadan, Rana Mahmoud

**Affiliations:** 1 Paediatric Department, Faculty of Medicine, Ain Shams University, Cairo, Egypt; 2 Clinical Pathology Department, Faculty of Medicine, Ain Shams University, Cairo, Egypt; 3 Clinical Pharmacy Practice Department, Faculty of Pharmacy, The British University in Egypt, Cairo, Egypt; 4 Pharmacology and Biochemistry Department, Faculty of Pharmacy, The British University in Egypt, Cairo, Egypt

**Keywords:** Obesity, Sphingolipids

## Abstract

**Background:**

Glucosylsphingosine (Lyso-GL-1), a glycosphingolipid formed by glucosylceramide hydrolysis, is known to be increased in Gaucher disease. Recently, increased ceramides and sphingolipids have been implicated in obesity, insulin resistance, and atherogenesis. However, limited data exists on serum Lyso-GL-1 level in children with obesity and its relation with insulin resistance, lipid dysfunction, and atherogenesis. Hence, this study aimed to assess Lyso-GL-1 level among children with obesity and correlate it with biomarkers of insulin resistance and atherogenic index of plasma (AIP).

**Methodology:**

Sixty children with obesity with a mean age of 10.06 years (SDS ± 2.22) and 60 age- and sex-matched normal-weighed controls were assessed for anthropometric measures, mean blood pressure percentiles, serum Lyso-GL-1, glycated hemoglobin (HbA1c), fasting insulin, triglycerides, cholesterol, low-density (LDL-C) and high-density lipoprotein cholesterol (HDL-C) with calculation of the homeostatic model assessment of insulin resistance (HOMA-IR) and the AIP.

**Results:**

Children with obesity have significantly higher Lyso-GL-1 and AIP than controls. Lyso-GL-1 is significantly positively correlated with body mass index (BMI) z-score, waist/hip ratio z-score, systolic and diastolic blood pressure percentiles, LDL-C, HOMA-IR, and AIP (*p* < 0.05), being independently correlated with systolic blood pressure percentile, LDL-C, and AIP on multivariate regression analysis.

**Conclusion:**

Serum Lyso-GL-1 is elevated in children with obesity, being closely correlated with hypertension, insulin resistance, and atherogenesis. This could provide a mechanistic insight on the role of Lyso-GL-1 in obesity and atherogenesis. Further studies are warranted to explore the potential role of Lyso-GL-1 as a biomarker and target for the prevention and treatment of obesity-related atherogenesis and insulin resistance.

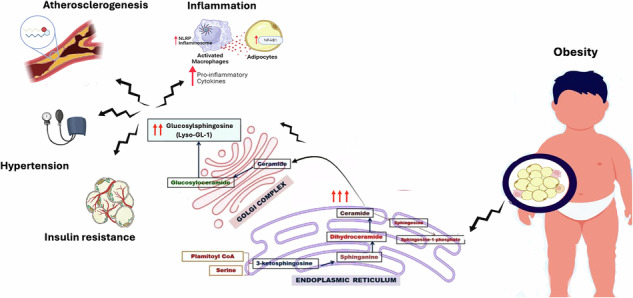

## Introduction

**Fig. 1:**
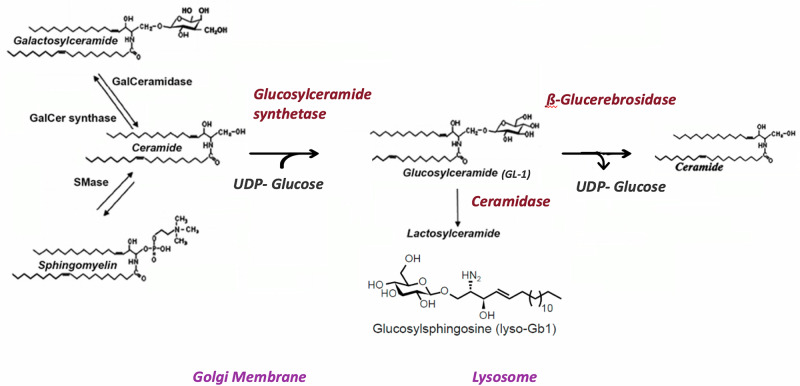
Metabolism of glucosylsphingosine and ceramides.

Glucosylsphingosine (also called Lyso-GL-1 or lyso-Gb-1) is a glucosphingolipid primarily formed by the deacylation of glucosylceramide by lysosomal acid ceramidase [1]. This process occurs when glucosylceramide accumulates in lysosomes, particularly within macrophages, and is then converted to glucosylsphingosine, Fig. 1. This conversion is accelerated when glucosylceramide accumulates due to deficiency in the enzyme ß-glucocerebrosidase, a hallmark of Gaucher disease [2, 3]. However, recent studies have demonstrated increased in Lyso-GL-1 in other conditions, including Parkinsonism, hematological diseases, and inflammation [4].

Recently, alterations in lysosphingolipid metabolism has been implicated in the pathogenesis of common metabolic conditions, including obesity and related metabolic disorders [5]. One of these sphingolipids is glucosylceramide; a glycosphingolipid and the precursor of glycosylsphingosine [6]. Adipocyte hypertrophy and hyperplasia in obesity lead to adipocyte dysfunction with accumulation of lipotoxic lipid metabolites and subsequent inflammasome activation, together with increased release of free fatty acids and cytokines [1]. In addition, lipid overloading of the adipocytes results in accumulation of ceramides and ceramide metabolites, including glucosylceramides, which, together with the pro-inflammatory state of adipose tissue, result in impaired insulin receptor signaling and metabolic derangements [7]. In vitro and human studies have shown that excessive consumption of fructose and glucose as in sugar-sweetened beverage is accompanied by significant alterion in the ceramides metabolism with accumulation of diacylglycerol, triacylgycerol and ceramides [8, 9]. In addition, pharmacological inhibition of sphingolipid synthesis was found to normalize ceramide profiles and improved sugar induced cardiometabolic risk [9]. Research suggests that increased levels of glucosylceramide and other sphingolipids in obesity contribute to insulin resistance and impaired adipocyte function. Inhibiting the synthesis of glucosylceramide can improve insulin sensitivity and reduce fat accumulation in animal models with obesity [10]. Meanwhile, ceramide accumulation in many metabolic tissues was found in obesity, causing numerous lipotoxic responses, including cell membrane dynamics modulation, endoplasmic reticulum and mitochondrial stress, and triggering an inflammasome in the endothelial cells and macrophages [11]. Moreover, elevated ceramides are increasingly recognized as a driver of atherosclerosis, being an independent risk factor for atherosclerotic cardiovascular disease, with ceramides, recently named the “second cholesterol,” being elevated in the blood and within the atherosclerotic plaques in people with cardiovascular diseases [12].

The atherogenic index of plasma (AIP) is a valuable tool composed of triglycerides (TG) and high-density lipoprotein cholesterol (HDL-C) that is commonly used as an indicator of dyslipidemia and cardiovascular risk [13]. Even in children, AIP was found to be closely associated with cardiovascular risk [14].

Hence, this study aimed to assess the serum level of glucosylsphingosine (Lyso-GL-1) among children with obesity and correlate it with waist/hip ratio standard deviation score (SDS) and homeostatic model assessment of insulin resistance (HOMA-IR) as indicators of insulin resistance, glycemic markers, namely glycated hemoglobin (HbA1c), and AIP as a biomarker of dyslipidemia and cardiovascular risk.

## Materials and methods

### Study population

Sixty children with obesity (Body mass index (BMI) ≥ 95th percentile, SDS ≥ 1.64) with a mean age of 10.06 years (SDS ± 2.22) were recruited from the Pediatric Diabetes and Endocrinology Unit, Pediatrics Hospital, Ain-Shams University during the period from January 2025 to April 2025, together with sixty age- and sex-matched healthy normal weighed siblings of children attending the outpatients clinic serving as controls (BMI between the 5th and 85th percentiles for age and sex) [15]. Participants were selected by simple random sampling. Exclusion criteria were family history of Gaucher disease or any hematological manifestations, presence of any cytopenia on complete blood picture, spleenegally by ultrasound, secondary obesity (e.g., Beckwith-Wiedemann, Prader-Willi), hypothyroidism, steroid-induced obesity, and the presence of comorbid chronic illnesses (e.g., diabetes mellitus).

Using the G*Power program for sample size calculation, setting power at 80% and alpha error at 5%, and assuming a medium effect size difference between children with obesity and normal-weight children (d = 0.3) regarding glucosylsphingosine level, based on this assumption, a sample size of at least 45 children with obesity and 45 normal-weight children will be needed.

### Ethical considerations

The study was approved by the Institutional Review Board (IRB) and the Research Ethics Committee of the Faculty of Medicine, Ain Shams University (FMASU REC), with an approval number of R 200/2021. Written informed consent was obtained from the legal guardians of each participant before enrollment after a full explanation of the study protocol. The study was done in line with the Consolidated Standards of Reporting Trials statement 2010 according to the Declaration of Helsinki [16].

### Study procedures

#### Clinical assessment

All participants participating in the study underwent:

(1) Complete history taking, including age, sex, age of onset of obesity, family history of obesity, and socioeconomic status assessed using the validated Arabic socioeconomic status scale for health research in Egypt. It is a scale with 7 domains with a total score of 84 [17].

(2) Physical examination including auxological assessment in the form of weight in kilograms (kg) using the Tanita scale, height in centimetres (cm) using the Harpendenstadiometer, and BMI in kg/m² with calculation of the SDS scores according to age and sex 10. Waist circumference was measured midway between the top of the iliac crest and the lowest rib, while hip circumference was measured in a horizontal plane at the extension of the buttocks, with calculation of the waist/hip ratio and comparison to normal references for age and sex according to Schwandt and colleagues till age 11 years and Mederico et al. above 11 years [18, 19].

Systolic and diastolic blood pressure were measured manually using a mercury sphygmomanometer two consecutive times in the right arm while the patient was relaxed and seated, with calculation of the average and plotting the results on the age- and sex-matched percentiles [20]. Tanner staging was used to assess sexual maturity [21].

#### Biochemical measurements

About 5 mL of venous blood were withdrawn from each participant in the morning after a 10-h fast and left for complete clotting; then serum was separated by centrifugation at 3000 rpm for 10 min and then stored at −20 °C for assessment of:

- Fasting blood glucose (intra- and inter-assay CVs, 2.3% and 3.5%; respectively) by Beckman Coulter AU 480 autoanalyzer (Beckman Coulter, Inc., 250 S. Kraemer Blvd., Brea, CA 92821, USA) and fasting insulin by immunometric, chemiluminescent assay on IMMULITE Autoanalyzer (Siemens Medical Solution Diagnostics, Los Angeles, USA).
- Insulin sensitivity was calculated using the HOMA-IR as follows: HOMA-IR = fasting glucose in millimoles per liter × fasting insulin in milli-international units per liter / 22.5. A value of >2.7 was the cutoffused as an index of insulin resistance in children and adolescents [22].
- Fasting serum TG (intra- and inter-assay CVs, 3.0% and 4.6%; respectively) and total cholesterol (TC) (intra- and inter-assay CVs, 2.8% and 4.2%, respectively) using quantitative enzymatic colorimetric technique by the Beckman Coulter AU 480 system (Beckman Coulter, Inc., 250 S. Kraemer Blvd., Brea, CA 92821, USA). Serum HDL-C (intra- and inter-assay CVs, 3.5% and 5.0%; respectively) by the phosphotungstate precipitation method (Bio Merieux kit, Marcyl’Etoile, Craponne, France). Low-density lipoprotein cholesterol (LDL-C) level was calculated using the Friedewald formula [23]. Dyslipidemia was defined according to the American academiy of pediatrics (AAP) revised consensus statement from the National Heart, Lung, and Blood Institute (NHLBI), using the following cut-off levels: TC ≥ 5.2 mmol/L, LDL-C ≥ 3.4 mmol/L, and HDL-C < 1.03 mmol/l with TG ≥ 1.47 mmol/L in children and adolescents 10-19 years of age and ≥ 1.13 mmol/L in children <10 years of age [24].
- AIP was calculated using the following formula: AIP = log10 (triglyceride/HDL cholesterol). A previously described cut-off of 0.27 in pediatrics was used as a predictor for cardiovascular risk [25].
- Lyso-GL-1 was assessed using commercially available Human Lyso-GL-1 ELISA kits supplied by Sunlong Biotech Co., Ltd, Hangzhou, China (Catalog No.: SL-3480Hu) following the manufacturer’s instructions. Absorbance of each well was measured at 450 nm by using a microtiter plate ELISA reader (Biotek, USA). with a detection range of 3 - 160 ng/L. Biochemical measurements were performed using validated in-house protocol. The coefficient of variation (CV) values were determined in consistent with the manufacturer’s specifications, the intra-assay coefficient of variation (CV) was <10%, and the inter-assay CV was <12%, based on replicate analyses performed in our laboratory.
- Another three mL of fresh whole blood were collected in EDTA and used for HbA1c analysis using turbidimetric inhibition immunoassay (TINIA) via the Tina-Quant® HbA1c kit supplied by Roche Diagnostics on the Cobas 6000 auto analyzer (Roche Diagnostics, GmbH, Mannheim, Germany) expressed in percentage.

### Statistical analysis

Data were collected, revised, coded, and entered into the Statistical Package for Social Science (IBM SPSS) version 27. The quantitative data were presented as mean, standard deviations, and ranges when their distribution was found to be parametric and median with interquartile range (IQR) when their distribution was found to be nonparametric. Also, qualitative data were presented as numbers and percentages. The comparison between groups with qualitative data was done by using the chi-square test, while the comparison between two independent groups with quantitative data and parametric distribution was done by using the independent *t*-test and with non-parametric distribution was done by using the Mann–Whitney test. Spearman correlation coefficients were used to assess the correlation between two quantitative parameters in the same group. Before multiple linear regression analysis, several variables were log-transformed to obtain (approximate) normal distribution. The multivariate analysis was adjusted for age, gender, height and socioeconomic scale. Simple regression analysis was first performed to screen potential associations for Lyso-GL-1, followed by a multivariate stepwise linear regression model to identify and determine significant associations for Lyso-GL-1 among children with obesity. Using the approach of stepwise variable selection, stepping up, only variables with a significance level of 0.05 were included in the model. The confidence interval was set to 95%, and the margin of error accepted was set to 5%. So, the p-value was considered significant at the level of < 0.05.

## Results

Sixty children with obesity with a median BMI SDS of 3.45 (IQR 3.18–3.93) and a mean age of 10.06 years (SDS ± 2.22) were compared to 60 age- and sex-matched normal-weighed children (*p* > 0.05). Fifty four of the studied children with obesity were non pubertal (90%), 48 had a Tanner stage of 1 (80%), 6 had a Tanner stage of 2 (10%) and 6 were Tanner stage 5 (10%). Regarding dyslipidemia; 20 children with obesity had hypertriglyceridemia (36.6%), 12 had decreased HDL-C (20%), 10 had elevated TC (16.7%) and 10 had elevated LDL-C (16.7%).

**Fig. 2:**
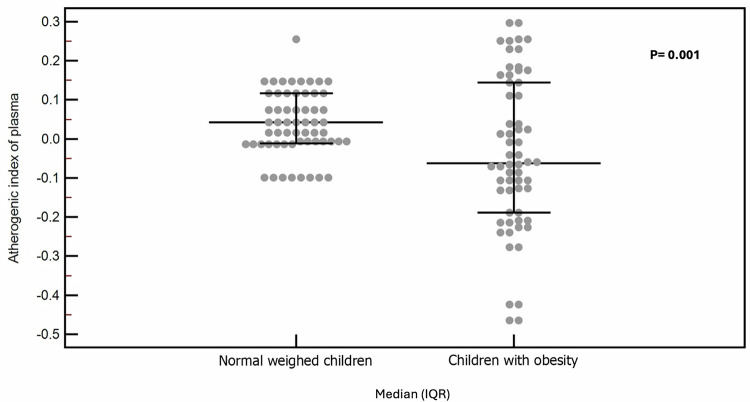
Comparison of atherogenic index of plasma among children with obesity and normal weighed children.

Children with obesity showed significantly higher waist/hip ratios, systolic and diastolic blood pressure percentiles, HOMA-IR and HbA1c than normal-weight children. Moreover, they had significantly higher TC, LDL-C, and AIP with significantly lower HDL-C than normal-weighed children, Table 1 and Fig. 2.

**Table 1:** Clinico-laboratory characteristics of the studied children with obesity and controls.

		Normal weighed children (*n* = 60)	Children with obesity (*n* = 60)	Test value	*P*-value
**Sociodemographic data**					
**Sex**	Males	26 (43.3%)	34 (56.7%)	2.133^a^	0.144
	Females	34 (56.7%)	26 (43.3%)		
**Age (years)**	Mean ± SD	9.70 ± 1.50	10.06 ± 2.22	1.040	0.300
	Range	7.19–12.25	5–15.4		
**Socio-economic scale**	Low	30 (50.0%)	20 (33.3%)	3.429^a^	0.064
	Middle	30 (50.0%)	40 (66.7%)		
	High	0 (0.0%)	0 (0.0%)		
**Clinical data**					
**Weight SDS**	Median (IQR)	−0.71 (−1.2 – −0.65)	3.12 (2.77 – 3.69)	−9.471^b^	**<0.001**
	Range	−1.36 – 0.91	1.56 – 5.06		
**Height SDS**	Median (IQR)	−0.77 (−1.05–0.08)	0.59 (−0.09 – 1.23)	−5.992^b^	**<0.001**
	Range	−1.53 – 1.4	−1.52 – 3.5		
**BMI SDS**	Median (IQR)	−0.11 (−1.17 – 0.26)	3.45 (3.18–3.93)	−9.464^b^	**<0.001**
	Range	−1.8–0.55	2.22 – 4.55		
**Waist circumference SDS**	Median (IQR)	−0.59 (−1.35 – −0.31)	5.36 (3.98 – 6.59)	−9.451^b^	**<0.001**
	Range	−1.66 – 0.55	1.3 – 45		
**Waist/ hip ratio SDS**	Median (IQR)	0.29 (−0.6–0.5)	1.88 (1.38 – 2.7)	−8.702^b^	**<0.001**
	Range	−1–1.4	−0.2 – 13.5		
**Systolic blood pressure percentile**	Mean ± SD	59.33 ± 17.06	88.37 ± 11.70	−10.870^c^	**<0.001**
	Range	50 – 90	53 – 100		
**Diastolic blood pressure percentile**	Mean ± SD	59.33 ± 17.06	81.46 ± 13.89	−7.790^c^	**<0.001**
	Range	50 – 90	37 – 100		
**Laboratory investigations**					
**Cholesterol (mmol/L)**	Mean ± SD	3.87 ± 0.50	4.30 ± 0.64	−4.142^c^	**<0.001**
	Range	3.18 – 4.60	3.26 – 5.85		
**HDL-C (mmol/L)**	Mean ± SD	1.21 ± 0.24	0.97 ± 0.15	−6.720^c^	**<0.001**
	Range	0.75 – 1.94	0.72 – 1.16		
**LDL-C (mmol/L)**	Mean ± SD	2.59 ± 0.50	2.79 ± 0.52	2.148^c^	**0.033**
	Range	1.78 – 3.21	1.55 – 4.10		
**Triglycerides (mmol/L)**	Mean ± SD	1.05 ± 0.12	1.15 ± 0.39	−1.926^c^	0.056
	Range	0.90 – 1.29	0.59 – 2.26		
**Fasting insulin (mIU/L)**	Median (IQR)	3.2 (2.5–3.68)	12 (9.25 – 15.64)	−9.205^b^	**<0.001**
	Range	0.91 – 7.2	5 – 41.8		
**Fasting glucose (mmol/L)**	Mean ± SD	4.51 ± 0.32	4.90 ± 0.36	−6.258^c^	**<0.001**
	Range	3.89 – 4.94	3.94 – 5.55		
**HOMA IR**	Median (IQR)	0.71 (0.55 – 0.82)	2.56 (1.98 – 3.64)	−9.420^b^	**<0.001**
	Range	0.18 – 1.25	1.17 – 10.11		
**HbA1C %**	Mean ± SD	4.53 ± 0.48	5.55 ± 0.34	−13.403^c^	**<0.001**
	Range	3.7–5.3	4.9 – 6.76		
**AIP**	Median (IQR)	0.03 (−0.01 – 0.12)	0.15 (−0.17 – 0.24)	−3.460^b^	**0.001**
	Range	−0.10–0.14	−0.16 – 0.29		
**Lyso-GL-1 (ng/L)**	Mean ± SD	7.10 ± 3.45	17.25 ± 7.34	−9.692^c^	**<0.001**
	Range	0 – 12	10 – 60		

*BMI* body mass index, *SDS* standard deviation score, *HDL* high density lipoproteins, *LDL−C* low density lipoproteins cholesterol, *HOMA−IR* homeostatic model of insulin resistance, *HbA1C* glycated hemoglobin, *AIP* atherogenic index of plasma; Lyso−GL-1: glucosylsphingosine.

*P* < 0.05: Significant (Bold).

^a^Chi-square test.

^b^Mann–Whitney test.

^c^Independent *t*-test.

### Glucosylsphingosine and obesity

**Fig. 3:**
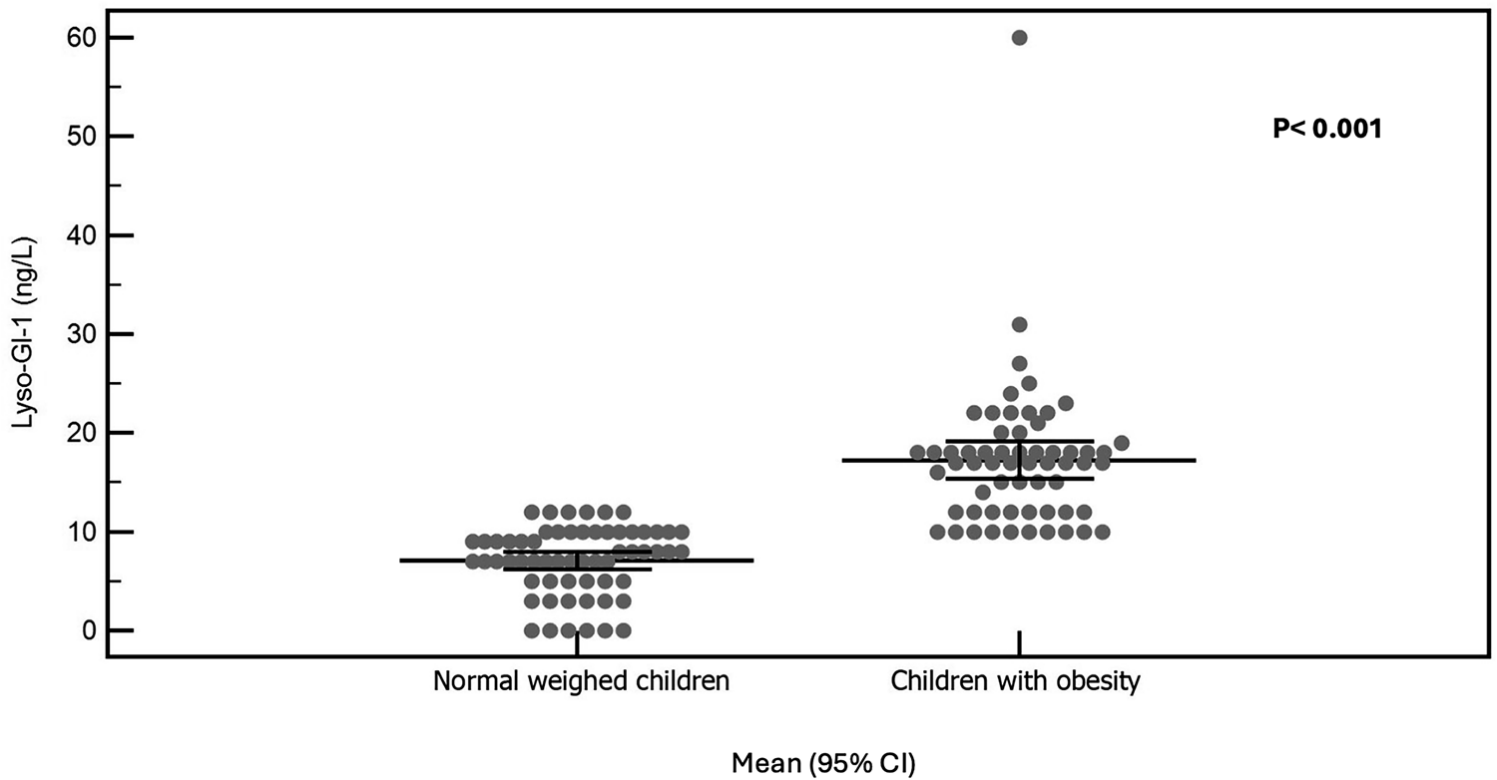
Comparison of serum Lyso-GL-1 level among children with obesity and normal weighed children.

**Fig. 4:**
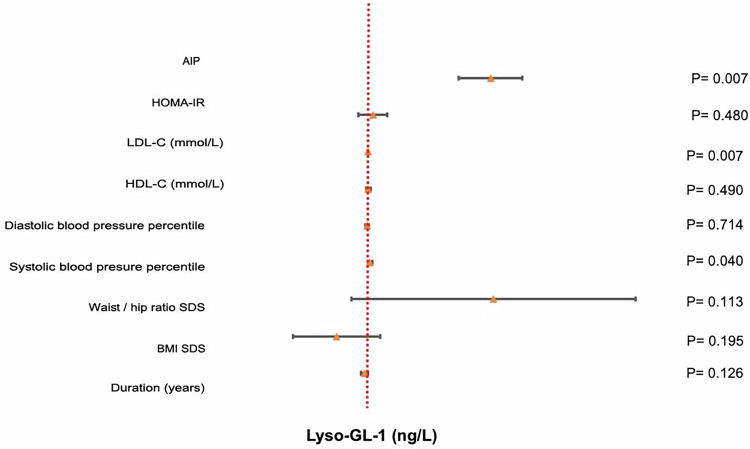
Forest plot for the multivariate linear regression analysis of factors associated with Lyso-GL-1 (ng/L) among the studied children with obesity.

The mean Lyso-GL-1 of the studied children with obesity was 17.25 ng/L, while that of normal-weight children was 7.10 ng/L (*p* < 0.01), Fig. 3. Lyso-GL-1 was found to be significantly correlated with BMI SDS and waist-hip ratio SDS (*p* < 0.01), Table 2. Regarding blood pressure, Lyso-GL-1 was found to be significantly correlated with both systolic and diastolic blood pressure percentiles (*p* < 0.001) among the studied children with obesity being independently associated with systolic blood pressure percentile using multivariate regression analysis (*p* = 0.040), Fig. 4. As for puberty, no significant relation was found between Lyso-GL-1 and Tanner staging (*p* = 0.569); however; this needs to be verified in further studies since the number of pubertal children in this study was small (only 6).

**Table 2:** Correlation between Lyso-Gl-1 and clinico-laboratory parameters among children with obesity.

	Lyso-Gl-1 (ng/L)			
	Children with obesity (*n* = 60)		Normal weighed children (*n* = 60)	
	*r*	*P*-value	*r*	*p*-value
Age (years)	−0.026	0.845	−0.119	0.363
Age of onset (years)	−0.009	0.947	N/A	N/A
Age at diagnosis (years)	−0.021	0.871	N/A	N/A
Weight SDS	−0.149	0.257	**0.319**	**0.013**
Height SDS	−0.144	0.272	−0.055	0.675
**BMI SDS**	**0.470**	**<0.001**	−0.015	0.907
Waist circumference SDS	−0.099	0.452	0.217	0.095
Hip circumference SDS	−0.179	0.256	0.011	0.97138
Waist/ hip ratio SDS	**0.379**	**<0.001**	−0.077	0.561
Systolic blood pressure percentile	**0.687**	**<0.001**	−0.069	0.600
Diastolic blood pressure percentile	**0.534**	**<0.001**	−0.069	0.600
Cholesterol (mmol/L)	0.173	0.185	0.050	0.707
HDL (mmol/L)	**−0.356**	**0.005**	0.037	0.778
LDL (mmol/L)	**0.706**	**<0.001**	0.060	0.650
Triglycerides (mmol/L)	0.207	0.112	−0.153	0.242
Fasting insulin (mIU/L)	−0.155	0.238	−0.313	0.450
Fasting glucose (mmol/L)	0.088	0.506	0.222	0.088
HOMA IR	**0.471**	**<0.001**	−0.202	0.122
HbA1C (%)	0.018	0.891	0.250	0.409
AIP	**0.848**	**<0.001**	0.018	0.893

Spearman correlation coefficient.

*BMI* body mass index, *SDS* standard deviation score, *HDL* high density lipoproteins, *LDL-C* low density lipoproteins cholesterol, *HOMA-IR* homeostatic model of insulin resistance, *HbA1C* glycated hemoglobin, *AIP* atherogenic index of plasma, *Lyso-Gl-1* glucosylsphingosine.

*P* < 0.05: Significant (Bold).

### Glucosylsphingosine and glycemic markers

Although, no significant correlation was found between serum Lyso-GL-1 level and HbA1c (*p* = 0.891); it was significantly correlated with HOMA-IR (*p* < 0.01), Table 2.

### Glucosylsphingosine, dyslipidemia, and atherogenesis

Worth mentioning, Lyso-GL-1 was found to be significantly correlated with LDL-C (*p* < 0.001), HDL-C (*p* = 0.05), and AIP (*p* < 0.01) among children with obesity but not correlated in normal weighed normal weighed children; Table 2; being independently associated with LDL-C (*p* = 0.007) and AIP (*p* = 0.007) among children with obesity on multivariate regression analysis; suggesting a possible role for Lyso-GL-1 in the development of dyslipidemia and atherogenesis; Fig. 4.

## Discussion

Obesity is characterized by a range of metabolic dysregulations, including insulin resistance, dyslipidemia, hypertension, and systemic inflammation, predisposing to atherogenesis and cardiovascular disease [26]. Despite the extensive research in that field, the exact pathophysiology of such metabolic derangements remains unclear.

Recently, alterations in the glucosylceramides metabolism are gaining attention being implicated in the pathophysiological consequences of obesity, including dyslipidemia, atherogenesis, and cardiometabolic diseases [27]. However, the role of Lyso-GL-1, a lysosphingolipid derivative of glucosylceramide, in obesity and obesity-related complications hasn’t been assessed.

In the current study, children with obesity were found to have significantly higher levels of Lyso-GL-1 than normal-weight children, with 83% of the studied children having Lyso-GL-1 levels above the previously described cut-off values [28]. This goes in line with Mamelli and colleagues, who demonstrated increased acid sphingomyelinase level (enzyme responsible for ceramides formation) in children with obesity [29]. This could be attributed to the chronic inflammatory state and altered lipid metabolism in obesity that contributes to increased turnover of complex sphingolipids, leading to elevated circulating lysolipids like Lysol-GL-1. Moreover, adipose tissue dysfunction in obesity can result in ectopic lipid accumulation and lysosomal stress, potentially enhancing sphingolipid degradation pathways [30]. In addition, increased levels of pro-inflammatory cytokines in obesity may upregulate glucosylceramide synthase and downstream sphingolipid intermediates, further contributing to Lysol-GL-1 accumulation [31]. This observed elevation of Lysol-GL-1 in children with obesity suggests a potential role for this bioactive sphingolipid in obesity-related metabolic dysregulation and highlights the need for identifying new cut-off values for Lyso-GL-1 in people with obesity.

Accumulating evidence suggests a role for glycosphingolipids, including glucosylceramides and their lysolipid counterparts, in insulin resistance through interfering with insulin signaling by activating pro-inflammatory pathways, inducing endoplasmic reticulum stress, and disrupting insulin receptor substrate function [32]. This goes in line with the current study, where a significant positive correlation was found between Lyso-GL-1 and HOMA-IR, suggesting an important role of Lyso-GL-1 in the pathophysiology of insulin resistance among children with obesity. In the same context, a murine study showed that inhibiting glycosphingolipid synthesis can significantly improve insulin sensitivity and glucose homeostasis, representing a novel therapeutic approach for insulin resistance [33].

In parallel, elevated levels of glycosphingolipids, including glucosylceramide, were found to contribute to dyslipidemia and atherogenesis by interfering with normal lipid trafficking and promoting lipid accumulation in tissues such as the liver and adipose tissue, thereby increasing circulating levels of atherogenic lipids [34]. This goes in concordance with the current study where Lyso-GL-1 was found to be significantly and independently correlated with LDL-C and AIP. Glycosphingolipids have been shown to promote atherogenesis through accumulation in the intima of atherosclerotic plaques and have been shown to exist there at levels higher than any other sphingolipid [35]. Glycosphingolipids do not exist unbound in the plasma but rather are associated with circulating lipoproteins, chiefly LDL-C [36]. Furthermore, glucosylceramide is the greatest inducer of pro-inflammatory cytokines in human coronary artery smooth muscle cells [35]. Indeed, inhibition of glucosylceramide synthesis in mice reduced inflammatory gene expression and atherosclerotic plaque formation [37]. Moreover, recent studies suggest that glucosylsphingosine accumulation promotes vascular inflammation, endothelial dysfunction, and macrophage foam cell formation, hallmarks of atherogenesis [38, 39]. Hence, Lyso-GL-1 may facilitate the progression of atherosclerotic lesions through enhancing oxidative stress and activating Toll-like receptor pathways, especially in the context of obesity and metabolic syndrome [40]. Collectively, these findings support a multifaceted role for glucosylsphingosine in linking lipid metabolism, insulin resistance, and atherogenic risk. Further studies are warranted to delineate whether elevated Lysol-GL-1 is a cause or consequence of obesity and to explore its utility as a predictive biomarker or therapeutic target in pediatric metabolic disorders.

## Strengths and limitations

This study is among the first to investigate serum Lyso-GL-1 in a pediatric population with obesity, exploring its pathomechanistic relationship with insulin resistance, dyslipidemia, and atherogenesis, providing a comprehensive assessment of the potential pathophysiological role of Lyso-GL-1 in atherogenesis.

However, the cross-sectional nature of the study limits its ability to establish causality between Lyso-GL-1 level and atherogenic outcomes. In addition, the young age and prepubertal state of most of the studied children limits it’s ability to verify it’s relation to puberty. Hence, further longitudinal studies with wider age range are warranted to explore whether Lyso-GL-1 is a potential target for prevention and treatment of dyslipidemia and atherogenesis in children with obesity.

## Conclusion

In conclusion, serum Lyso-GL-1 is elevated in children with obesity; this elevation is closely linked to insulin resistance and dyslipidemia. Hence, Lyso-GL-1 may serve as an early biomarker of dyslipidemia and vasculopathy, suggesting the need to further elucidate the potential role for Lyso-GL-1 in the prevention and management of dyslipidemia and atherogenesis among children and adolescents with obesity.

## Data Availability

Data will be available from the corresponding author upon reasonable request.
